# Molecular-based detection of *Ehrlichia* spp. in stray dogs-infesting *Rhipicephalus* ticks in high-altitude of northern Pakistan

**DOI:** 10.1371/journal.pone.0342091

**Published:** 2026-03-06

**Authors:** Abdul Majid, Muhammad Numan, Hadia Tila, Iram Liaqat, Mohibullah Shah, Zhihua Sun, Abid Ali, Mashal M. Almutairi

**Affiliations:** 1 Department of Zoology, Abdul Wali Khan University Mardan, Mardan, Khyber Pakhtunkhwa, Pakistan; 2 Microbiology Lab, Department of Zoology, Government College University, Lahore, Punjab, Pakistan; 3 Department of Biochemistry, Bahauddin Zakariya University, Multan, Pakistan; 4 College of Animal Science and Technology, Shihezi University, Shihezi, China; 5 Department of Pharmacology and Toxicology, College of Pharmacy, King Saud University, Riyadh, Saudi Arabia; University of Minnesota, UNITED STATES OF AMERICA

## Abstract

Although ticks are recognized as carriers of zoonotic pathogens, the risks of tick-borne infections associated with dogs have received limited attention. The close interaction between humans and dogs increases the zoonotic potential of pathogens, increasing the urgency of addressing this overlooked vector-borne health risk, especially in developing regions. This study sought to identify tick-borne zoonotic pathogens by collecting ticks from dogs and screening for *Ehrlichia* species, a group known to pose significant yet often neglected health risks to both humans and animals, particularly in the northern area of Khyber Pakhtunkhwa (KP), Pakistan. Ticks were collected from dogs in the Mardan and Dir Lower districts of Pakistan from June 2024 to May 2025. The collected ticks were morphologically identified and processed for molecular identification using 16S ribosomal DNA (16S rDNA) and cytochrome c oxidase (*cox1*) sequences for ticks, and 16S rRNA and *dsb* sequences were used for their associated *Ehrlichia* spp. Among the 223 dogs observed, 147 were infested with ticks, resulting in an overall prevalence of 66%. *Rhipicephalus* ticks’ infestation were more common in female dogs (91/113, 80.53%) than in male dogs (56/110, 51%). The occurrence of adult females was highest (173/432, 40%), followed by males (158/432, 36.57%) and nymphs (101/432, 23.37%). Two tick species were identified: *Rhipicephalus haemaphysaloides* and *Rhipicephalus* sp., which was identified as a member of the *Rhipicephalus sanguineus* species complex. This was confirmed by 16S rDNA and *cox1* sequences, which showed 99–100% maximum identity. In the phylogenetic trees, *Rh. haemaphysaloides* clustered with the same species reported from Pakistan, while *Rhipicephalus* sp. grouped with *Rhipicephalus* sp. morphotype III from Pakistan and India. Regarding their associated bacterial species, such as *Ehrlichia* spp., they were detected in both *Rhipicephalus* sp. and *Rh. haemaphysaloides* based on 16S rDNA and dsb sequences, which revealed maximum identity with *Ehrlichia minasensis* and *Ehrlichia* spp., respectively. These species phylogenetically clustered with the corresponding *Ehrlichia* species. The high infection rate observed in ticks suggests a significant relationship between companion animals and pathogen vectors. Further studies are necessary to investigate the potential health risks of tick-borne *Ehrlichia* spp. and their zoonotic implications in the region.

## Introduction

Ticks (Acari: Ixodidae) are obligatory blood-sucking parasites found on all terrestrial and semitrestrial vertebrates and vectors of pathogens such as viruses, bacteria, and protozoans [1,2]. Ticks transmit a diverse range of pathogens, including *Ehrlichia*, *Rickettsia*, *Anaplasma, Coxiella*, *Theileria*, *Babesia*, and *Borrelia* species [3,4,5,6,7].

*Ehrlichia* spp*.* (Rickettsiales: Anaplasmataceae) are gram-negative intracellular alpha proteobacteria that are transmitted primarily by ticks and are responsible for causing ehrlichiosis, an important infectious disease affecting both humans and animals [8]. The genus *Ehrlichia* includes several well-known species, such as *Ehrlichia canis, Ehrlichia chaffeensis, Ehrlichia ruminantium, Ehrlichia muris, Ehrlichia ewingii, Ehrlichia minasensis* and *Ehrlichia ovina* [9,10]. Studies in recent years have revealed additional species and candidate taxa, including *Ehrlichia* spp*.* Yonaguni138 and *Ehrlichia* spp*.* Baoji96*,* reflecting the continuous discovery and genetic complexity of this genus [11,12]*. Ehrlichia chaffeensis* was identified as the primary species causing human ehrlichiosis, whereas *E. ewingii* and *E. muris* have been reported to cause human infections in North America [13,14]. Tick species from genera such as *Amblyomma, Dermacentor, Hyalomma, Ixodes*, and *Rhipicephalus* are known to transmit *Ehrlichia* spp*.* [15,4,16,17,18].

Canine monocytic ehrlichiosis (CME) is widely recognized as the primary tick-borne zoonotic disease affecting dogs worldwide [19]. Dogs are the main hosts for *E. canis*, which is spread by the brown dog tick *Rh. sanguineus*. This tick is uniquely adapted to environments where dogs live, such as homes, kennels, and outdoor spaces, facilitating its life cycle and the continuous spread of *E. canis* [20]. In addition to being susceptible to infection, dogs are critical in perpetuating *E. canis* in tick populations, which significantly impacts disease epidemiology [8,21]. This close vector relationship enhances the risk of ehrlichiosis transmission, especially in tropical and subtropical regions where *Rh. sanguineus* thrives [22]. The brown dog ticks *Rh. sanguineu*s is primarily responsible for transmitting the etiological agent of CME, thereby facilitating the spread of the disease [10]; hence, the pathogens are more prevalent in areas where brown dog tick distribution is ubiquitous [22]. The close bonding relationship between humans and dogs puts them at risk of contracting these infections [19,23]. Some *Ehrlichia* spp*.* have been reported as the cause of human ehrlichiosis, and the zoonotic behavior of human ehrlichiosis is supported by the fact that the same *Ehrlichia* spp*.* also infect rodents (*Apodemus speciosus*), deer (*Odocoileus virginianus*), horses (*Equus caballus*), and dogs (*Canis familiaris*) [24,25].

The brown dog ticks *Rh. sanguineus* sensu lato (s.l.) and *Rh. haemaphysaloides* are more common in tropical and subtropical areas of the world and can be found in both rural and urban areas [26]. Research has indicated that *Rh. sanguineus* s.l. is not a single species but rather a species complex comprising distinct genetic groups, known as temperate and tropical lineages. The temperate lineage is typically associated with *Rh. sanguineus* sensu stricto (s.s.), whereas the tropical lineage includes *Rh. linnaei* and other cryptic species [27]. In Pakistan, reports of *Rh. sanguineus* most likely represent closely related species rather than the true *Rh. sanguineus* s.l. and, owing to their morphological resemblance*, Rh. turanicus* has often been confused with *Rh. sanguineus*: however, molecular research has confirmed that it is a separate species [28,29].

In Pakistan, where tick-borne pathogens are prevalent, these pathogens pose significant threats to livestock holders [1,30,31,32,6,33,32]. Various studies have reported the presence of important tick-borne pathogens in Pakistan, including *Anaplasma* spp., *Ehrlichia* spp., *Rickettsia* spp., *Babesia* spp., *Theileria* spp., and *Borrelia* spp., in different ticks that infest various animal hosts [30,1,9,31,34]. However, limited molecular data are available on *Ehrlichia* spp*.*, specifically in ticks collected from dogs in Khyber Pakhtunkhwa (KP), Pakistan. Therefore, this study aimed to identify ticks that infest dogs and evaluate the occurrence of *Ehrlichia* spp*.* in collected ticks.

## Materials and methods

### Ethical approval

The current study was approved by the Advance Studies Research Board (ASRB): Dir/A&R/AWKUM/2023/00141) committee members of Abdul Wali Khan University Mardan, Khyber Pakhtunkhwa, Pakistan (Approval No. Dir/A&R/AWKUM/2023/00141). Oral permission was obtained from the dog owners or caretakers when applicable for observation and tick collections. All the specimens were collected in publicly accessible areas, and no private or protected land was accessed. No endangered or protected species were involved in this study.

### Study area

**Fig 1:**
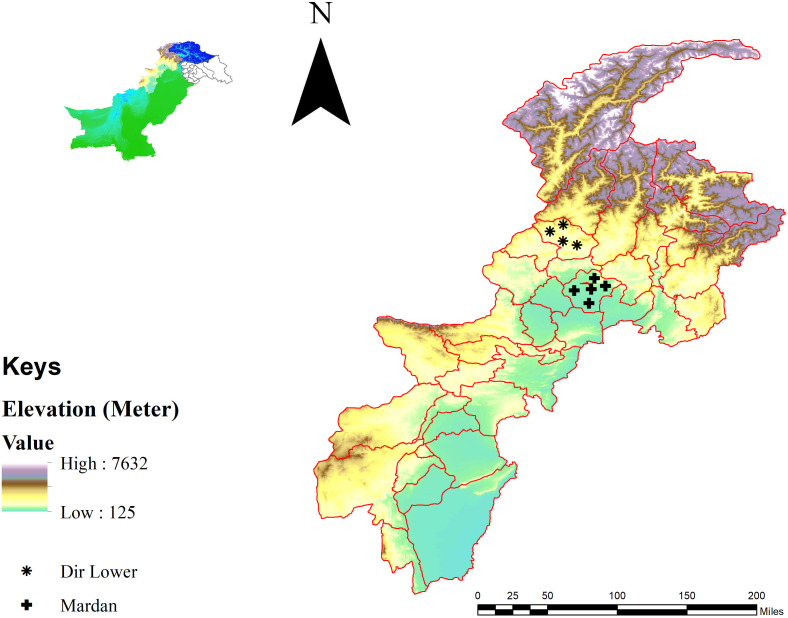
Map showing collection sites of ticks in different areas of the selected districts.

The current research was conducted in two districts of KP, Pakistan: Mardan (34°11’54.6”N 72°01’37.4”E) and Dir Lower (34°52’12.1”N 71°49’00.8”E). District Mardan is located in the plains, whereas Dir Lower lies in comparatively mountainous area. The average winter temperatures (December to February) range between 10–17℃ in Mardan and in 9–15℃ in Dir Lower, while in summer (June to September) temperatures rise to 17–40℃ in Mardan and 15–35℃ in Dir Lower 15–35℃, respectively [1]. A global positioning system (GPS) was used to determine the geographical coordinates of the collection sites, and a study map was designed via ArcGIS v. 10.3.1 (ESRI, Redlands, CA, United States) based on publicly availble administrative boundary shapefile obtained from DIVA-GIS (Global Administrative Areas shapefiles; https://diva-gis.org/data.html) (Fig 1).

### Tick collection and morphological identification

Ticks were collected via convenience sampling from stray dogs in different localities of chosen districts (Mardan and Dir Lower) of Pakistan from June 2024 to May 2025. All tick specimens (males, females, and nymphs) were collected whenever stray dogs were found, regardless of the specific location or time, including streets, marketplaces, and other areas where they roamed freely. The specimens were carefully removed from the host body using tweezers to prevent any external damage. The collected tick specimens were washed with distilled water, followed by 70% ethanol and stored in properly labeled 1.5 ml microtubes. Ticks were immediately preserved in 100% ethanol for further molecular analysis. The collected ticks were morphologically identified via standard morpho-taxonomic features [22,28,35] under a stereozoom microscope (SZ61, Olympus Corporation, Tokyo, Japan).

### DNA extraction and PCR

Total 119 (15 males, 54 females, and 50 nymphs) morphologically identified ticks were subjected to DNA extraction. Before extraction, the tick samples were rinsed with distilled water, followed by 70% ethanol, and kept in an incubator until drying. Whole ticks were cut with sterile scissors and homogenized in phosphate-buffered saline (PBS) via a micropestle for DNA extraction via the Phenol chloroform protocol [36]. A NanoDrop instrument (Nano-Q, Optizen, Daejeon, South Korea) was used for the quantification of double-stranded DNA, and the samples were stored at −20°C for further analysis.

16S ribosomal DNA (16S rDNA) and cytochrome C oxidase (*cox1*) fragments were amplified for the molecular identification of ticks via conventional PCR (GE-96G, BIOER, Hangzhou, China). Each PCR mixture contained 25 µl of each primer (10 µM), 8.5 µl of PCR “nuclease-free” water, 12.5 µl of Dream*Taq* Green MasterMix (2x) (Thermo Scientific, Waltham, MA, USA) and 2 µl of template DNA (50 ng).

The extracted DNA was used for the screening of *Ehrlichia* spp*.* targeting the 16S rDNA and dsb gene. Each PCR experiment contained negative control, in which PCR grade water was used instead of template DNA and a positive control *Rh. microplus* DNA for tick samples and *Anaplasma marginale* DNA for *Ehrlichia* spp*.* The primers used in this study are listed in Table 1. The thermocycler conditions used were previously described [38,40,39,37]. The PCR products were electrophoresed on a 1.5% agarose gel, and the amplified samples were visualized under UV light through gel documentation system (BioDoc-IT^TM^ Imaging systems UVP, LLC, Upland, USA).

**Table 1:** List of primers used for the amplification of various genes fragments in the present study.

Organism/Gene	Primer	Sequence	Amplicon size	References
Tick/ 16S	16S+1 16S-1	CCGGTCTGAACTCAGATCAAGT GCTCAATGATTTTTTAAATTGCTGT	460 bp	[37]
Tick/ *cox1*	cox1 F cox1 R	GGAACAATATATTTAATTTTTGG ATCTATCCCTACTGTAAATATATG	850 bp	[38]
*Ehrlichia* spp./ 16S rDNA	EHR16SD EHR16SR	GGTACCYACAGAAGAAGTCC TAGCACTCATCGTTTACAGC	345 bp	[39]
*Ehrlichia* spp. Dsb	DSB-330 F DSB-380 F DSB-720 R	GATGATGTTTGAAGATATSAAACAAAT ATTTTTAGRGATTTTCCAATACTTGG CTATTTTACTTCTTAAAGTTGATAWATC	349 bp	[40]

### DNA sequencing and phylogenetic analysis

All positive products of 16S rDNA and *cox1* for ticks, as well as 16S rRNA and *dsb* for associated *Ehrlichia* spp*.*, were sequenced bidirectionally (Macrogen Inc., Seoul, South Korea) using the Sanger sequencing method. The obtained sequences were trimmed using SeqMan V. V. 5 (DNASTAR, Inc., Madison/WI, USA) to remove poor-quality reads and primer contamination, then subjected to BLAST (Basic Local Alignment Search Tool) at NCBI (National Center for Biotechnology Information) [41]. Sequences with maximum identities were downloaded in FASTA format from NCBI. These sequences were aligned together with the outgroup and newly obtained sequences using BioEdit V. 7.0.5 [42]. The phylogenetic trees were constructed for tick 16S rDNA and *cox1* sequences, as well as for *Ehrlichia* 16S rDNA and *dsb* sequences, using Bayesian Inference (BI) in MrBayes v. 3.2.7 [43], accessed via the NGPhylogeny server (https://ngphylogeny.fr/) [44]. The nucleotide datasets were analyzed with the General Time Reversible (GTR) model, followed by Markov Chain Monte Carlo (MCMC) analysis run for 100,000 generations with 4 chains and a single run [45]. Tree and model parameters were sampled every 500 generations [43]. The consensus tree was derived from post-burn-in samples, with Bayesian posterior probabilities indicating branch support [45]. The resulting tree files were subjected to the iTOL server (https://itol.embl.de/) for visualization [46].

## Results

### Description of dog ticks

**Fig 2:**
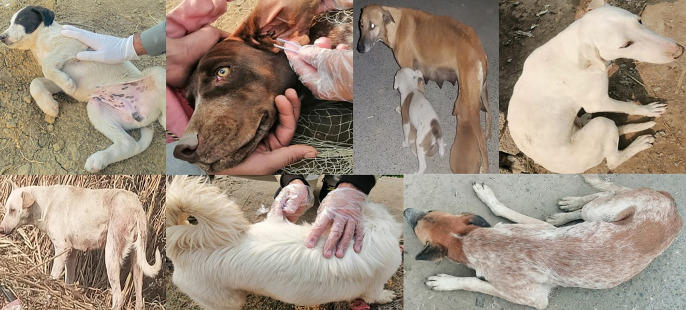
Collection of *Rhipicephalus* tick species from stray dogs in this study from district Mardan and Dir Lower.

A total of 223 dogs were observed in two districts (Fig 2), Mardan (112) and Dir Lower (111), for tick collection from June 2024 to May 2025. was Among these, 147/223 (66%) dogs were infested with *Rhipicephalus* ticks, and the burden of ticks was higher on female dogs (91/113, 80.53%) than in male dogs (56/110, 51%). The occurrence of ticks was high in the district of Mardan (236/432, 54.62%), followed by Dir Lower (196/432, 45.37%), morphologically identified as two species: *Rhipicephalus* sp. (within *Rhipicephalus sangunieus* species complex) and *Rh. haemaphysaloides*. The highest occurrence was *Rhipicephalus* sp. 66.89% (289/432: 122 females, 108 males and 59 nymphs), followed by *Rh. haemaphysaloides* 33.33% (144/432: 51 females, 50 males and 42 nymphs). Among the collected ticks, the percentage of females was greater (173/432, 40%) than that of males (158/432, 36.57%) and nymphs (101/432, 23.37%) (Table 2).

**Table 2:** Spatial and host distribution of ticks infesting dogs and molecular detection of *Ehrlichia* spp. in Khyber Pakhtunkhwa.

Districts	Locations	Host details				Identified tick species	Collected ticks				Molecularly analyzed ticks	Detection of *Ehrlichia* spp.
		Gender	Observed	Infested (%)	Chi-square (p value)		Female (%)	Nymph (%)	Male (%)	Total (%)		
Mardan	Mardan	Male	8	3 (2.0)	1.64 (0.19)	*Rhipicephalus* sp.	6 (1.38)	3 (0.69)	4 (0.92)	13 (3.0)	5 (2F, 3N)	1F
						*Rh. haemaphysaloides*	3 (0.69)	4 (0.92)	4 (0.92)	11 (2.54)	3 (1F, 2N)	—
		Female	12	8 (5.4)	0.26 (0.60)	*Rhipicephalus* sp.	5 (1.15)	3 (0.69)	2 (0.46)	10 (2.31)	2 (1F, 1N)	1N
						*Rh. haemaphysaloides*	3 (0.69)	1 (0.23)	3 (0.69)	7 (1.62)	3 (2F, 1N)	—
	Takht Bhai	Male	10	7 (4.8)	0.07 (0.78)	*Rhipicephalus* sp.	3 (0.69)	2 (0.46)	4 (0.92)	9 (2.08)	3 (2F, 1N)	—
						*Rh. haemaphysaloides*	2 (0.46)	3 (0.69)	3 (0.69)	8 (1.85)	5 (1M, 3F, 1N)	—
		Female	11	8 (5.4)	0.26 (0.60)	*Rhipicephalus* sp.	8 (1.85)	2 (046)	9 (2.08)	19 (4.39)	4 (1M, 2F, 1N)	—
						*Rh. haemaphysaloides*	3 (0.53)	3 (0.69)	3 (0.69)	9 (1.08)	3 (2F, 1N)	1N
	Katlang	Male	9	5 (3.4)	2.47 (0.11)	*Rhipicephalus* sp.	8 (1.85)	7 (1.62)	7 (1.62)	22 (4.86)	5 (1M, 3F, 1N)	—
						*Rh. haemaphysaloides*	3 (0.69)	1 (0.23)	2 (0.46)	6 (1.38)	2 (1F, 1N)	—
		Female	13	9 (6.1)	0.00 (1.00)	*Rhipicephalus* sp.	9 (2.08)	4 (0.92)	6 (1.38)	19 (4.39)	2 (1F, 1N)	1F
						*Rh. haemaphysaloides*	4 (0.92)	4 (0.92)	2 (0.46)	10 (2.31)	3 (1F, 2N)	—
	Rustam	Male	10	7 (4.8)	1.23 (0.26)	*Rhipicephalus* sp.	5 (1.15)	4 (0.92)	4 (0.92)	13 (3.0)	3 (1F, 2N)	—
						*Rh. haemaphysaloides*	2 (0.46)	3 (0.69)	3 (0.69)	8 (1.85)	5 (1M, 2F, 2N)	—
		Female	15	11 (7.5)	0.00 (1.00)	*Rhipicephalus* sp.	5 (1.15)	2 (0.46)	4 (0.92)	11 (2.54)	4 (1M, 1F, 2N)	—
						*Rh. haemaphysaloides*	2 (0.46)	2 (0.46)	3 (0.69)	7 (1.62)	2 (1F, 1N)	—
	Lund Khwar	Male	8	3 (2.0)	0.42 (0.51)	*Rhipicephalus* sp.	6 (1.38)	3 (0.69)	3 (0.69)	12 (2.77)	5 (2M, 1F, 2N)	—
						*Rh. haemaphysaloides*	3 (0.69)	2 (0.46)	4 (0.92)	9 (2.08)	2 (1M, 1N)	1N
		Female	16	13 (8.8)		*Rhipicephalus* sp.	10 (2.31)	4 (0.92)	9 (2.08)	23 (5.32)	4 (1F, 3N)	—
					1.64 (0.19)	*Rh. haemaphysaloides*	4 (0.92)	3 (0.69)	3 (0.69)	10 (2.31)	3 (1F, 2N)	1F
Dir Lower	Adenzai	Male	18	9 (6.1)	0.26 (0.60)	*Rhipicephalus* sp.	9 (2.08)	4 (0.92)	5 (1.15)	18 (4.16)	2 (2F)	—
						*Rh. haemaphysaloides*	3 (0.69)	3 (0.69)	2 (0.46)	8 (1.85)	2 (1M, 1F)	1F
		Female	12	11 (7.5)	0.07 (0.78)	*Rhipicephalus* sp.	6 (1.38)	3 (0.69)	11 (2.54)	20 (4.62)	3 (2F, 1N)	—
						*Rh. haemaphysaloides*	2 (0.46)	2 (0.46)	3 (0.69)	7 (1.62)	2 (1F, 1N)	—
	Lal Qilla	Male	17	7 (4.8)	0.26 (0.60)	*Rhipicephalus* sp.	8 (1.85)	3 (0.69)	7 (1.62)	18 (4.16)	4 (1M, 2F, 1N)	1N
						*Rh. haemaphysaloides*	4 (0.92)	3 (0.69)	2 (0.46)	9 (2.08)	2 (1F, 1N)	—
		Female	11	10 (6.8)	2.47 (0.11)	*Rhipicephalus* sp.	8 (1.85)	3 (0.69)	5 (1.15)	16 (3.70)	3 (1M, 1F, 1N)	—
						*Rh. haemaphysaloides*	3 (0.69)	1 (0.23)	2 (0.46)	6 (1.38)	2 (1F, 1N)	—
	Samarbagh	Male	14	8 (5.4)	0.00 (1.00)	*Rhipicephalus* sp.	6 (1.38)	2 (0.46)	8 (1.85)	16 (3.70)	5 (2F, 3N)	—
						*Rh. haemaphysaloides*	2 (0.46)	1 (0.23)	3 (0.69)	6 (1.38)	2 (1F, 1N)	1N
		Female	13	12 (8.2)	1.23 (0.26)	*Rhipicephalus* sp.	6 (1.38)	3 (0.69)	7 (1.62)	16 (3.70)	3 (1F, 2N)	—
						*Rh. haemaphysaloides*	3 (0.69)	3 (0.69)	3 (0.69)	9 (2.08)	6 (1M, 3F, 2N)	1F
	Timergara	Male	16	7 (4.8)	0.00 (1.00)	*Rhipicephalus* sp.	6 (1.38)	4 (0.92)	5 (1.15)	15 (3.67)	2 (1F, 1N)	—
						*Rh. haemaphysaloides*	2 (0.46)	1 (0.23)	3 (0.69)	6 (1.38)	6 (1M, 3F, 2N)	—
		Female	10	9 (6.1)	0.42 (0.51)	*Rhipicephalus* sp.	8 (1.85)	3 (0.69)	8 (1.85)	19 (4.39)	2 (1M, 1N)	—
						*Rh. haemaphysaloides*	3 (0.69)	2 (0.46)	2 (0.46)	7 (1.62)	5 (1M, 3F, 1N)	—
Overall		**Male**	**110**	**56 (38.1)**		***Rhipicephalus* sp.**	**57 (13.19)**	**17 (3.93)**	**48 (11.11)**	**122 (28.24)**	**34 (4M, 16F, 14N)**	**3 (2F, 1N)**
						***Rh. haemaphysaloides***	**24 (5.55)**	**13 (3.0)**	**26 (6.01)**	**63 (14.58)**	**29 (5M, 13F, 11N)**	**2 (1F, 1N)**
		**Female**	**113**	**91 (61.9)**		***Rhipicephalus* sp.**	**65 (15)**	**42 (9.72)**	**61 (14.12)**	**168 (38.88)**	**27 (4M, 10F, 13N)**	**2 (1F, 1N)**
						***Rh. haemaphysaloides***	**27 (6.25)**	**30 (6.94)**	**24 (5.55)**	**81 (18.75)**	**29 (2M, 15F, 12N)**	**2 (1F, 1N)**
		**Total**	**223**	**147 (100)**			**173 (40.0)**	**101 (23.61)**	**158 (36.57)**	**432 (100)**	**119 (15M, 54F, 50N)**	**9 (5F, 4N) (7.56%)**

### Sequencing analysis and phylogeny of ticks

**Fig 3:**
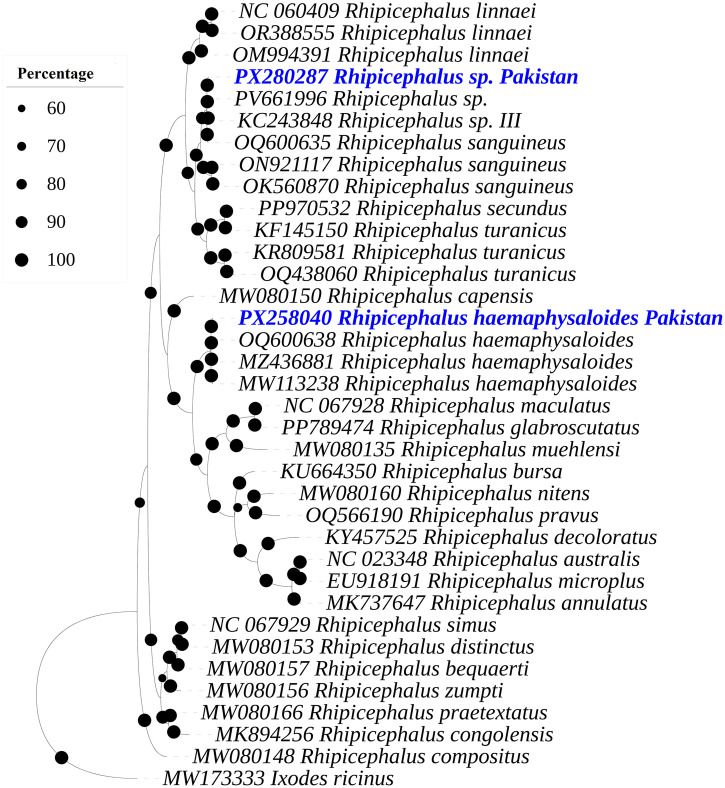
A Bayesian Inference phylogenetic tree was constructed on the basis of 16S rDNA sequences of *Rhipicephalus* spp. The *Ixodes ricinus* 16S rDNA sequence was used as an outgroup. The bootstrap supporting values (60-100%) are indicated at each node. The obtained sequences are represented in blue font and accession number PX280287 (*Rhipicephalus* sp.) and PX258040 (*Rhipicephalus haemaphysaloides*).

The obtained sequences of 16S rDNA from morphologically identified ticks such as *Rh. haemaphysaloides* and *Rhipicephalus* sp. were 386 bp and 390 bp size, respectively. The BLAST analysis of the 16S rDNA fragment of *Rhipicephalus* sp. revealed 99.74–100% maximum identity with *Rhipicephalus* sp., *Rhipicephalus* sp. morphotype III and *Rh. sanguineus* from Pakistan. Phylogenetic analysis further revealed that the obtained 16S rDNA sequences for *Rhipicephalus* sp. clustered with those of *Rhipicephalus* sp. (PV661996), *Rhipicephalus* sp. morphotype III from Pakistan (KC243848) and the previously reported *Rh. sanguineus* reported from the same region (ON921117, OK560870, and OQ600635). While the BLAST analysis of the 16S rDNA sequences of *Rh. haemaphysaloides* revealed 99.73–100% identity with same species sequences reported from Pakistan. In the phylogenetic tree, *Rh. haemaphysaloides* clustered with the corresponding sequences from Pakistan (MZ436881, OQ600638 and MW113238) (Fig 3).

**Fig 4:**
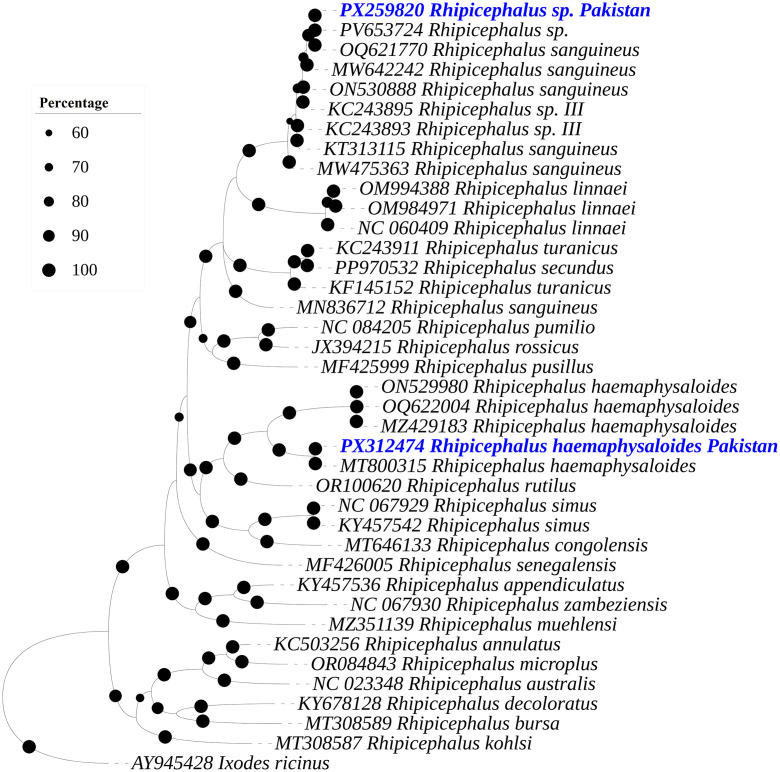
A Bayesian Inference phylogenetic tree was constructed on the basis of *cox1* sequences of *Rhipicephalus* spp. The *Ixodes ricinus cox1* sequence was used as an outgroup. The bootstrap supporting values (60-100%) are indicated at each node. The obtained sequences are represented in blue font and accession number PX259820 (*Rhipicephalus* sp.) and PX312474 (*Rhipicephalus haemaphysaloides*).

The size of the trimmed obtained sequences for *cox1* was 608 bp for *Rhipicephalus* sp. and 604 bp for *Rh. haemaphysaloides*. BLAST analysis revealed that the *cox1* sequence of *Rhipicephalus* sp. showed 99.72–100% identity with *Rhipicephalus* sp. or *Rhipicephalus* sp. morphotype III, and *Rh. sanguineus*, whereas the *Rh. haemaphysaloides* sequence showed 100% identity with the same species reported from Pakistan. In the phylogenetic tree, the *Rhipicephalus* sp. sequence clustered with the sequences of *Rhipicephalus* sp. (PV653724 from Pakistan) or *Rhipicephalus* sp. morphotype III (KC243893 from India, and KC243895 from Pakistan), and *Rh. sanguineus* (MW642242, ON530888 and OQ621770 from Pakistan), while *Rh. haemaphysaloides* sequence grouped with corresponding species sequences reported from Pakistan (OQ622004, MZ429183, ON529980, and MT800315) (Fig 4).

The obtained 16S rDNA and *cox1* sequences, as revealed by the BLAST results, had the highest identity with the species identified as *Rh. sanguineus* from Pakistan; however, the sequences for this species most likely represent a different species within the *Rh. sanguineus* species complex rather than *Rh. sanguineus*.

The obtained 16S rDNA sequences were submitted to GenBank under the accession numbers PX258040 (*Rh. haemaphysaloides*) and PX280287 (*Rhipicephalus* sp.); *cox1* sequences under the accession numbers PX312474 (*Rh. haemaphysaloides*) and PX259820 (*Rhipicephalus* sp.).

### Detection of *Ehrlichia* spp. in Ticks

A total of 119 tick DNA samples were screened for the detection of *Ehrlichia* spp*.* Overall, 7.56% (9/119) of the ticks were positive. Both reported tick species, *Rhipicephalus* sp. and *Rh. haemaphysaloides* were positive for *Ehrlichia* spp*.*, with detection rates of 8.1% (5/61) and 6.89% (4/58), respectively. The prevalence of *Ehrlichia* spp. in the district of Mardan (five sampling sites) was 7.35% (05/68), and that in the Dir Lower (four sampling sites) was 7.84% (04/51) (Table 2).

The BLAST results of the 16S rRNA sequence (309 bp) of *Ehrlichia* sp. from *Rhipicephalus* sp. and *Rh. haemaphysaloides* showed 100% maximum identity with *Ehrlichia* sp. reported from Pakistan (MH250197, ON926911, ON926912 and OQ616507) and China (KJ410254 and JX402603). While other 16S rRNA (303 bp) sequence from the same hosts showed 100% maximum identity with *E. minasensis* reported from Turkey (OQ592787), China (PQ896500, OQ136683, OQ506483 and OQ136684), Kenya (MT163431 and MT163429), Pakistan (OQ616505) and Australia (MH500005).

The BLAST results of the *dsb* (340 bp) of *Ehrlichia* sp. sequence from *Rhipicephalus* sp. and *Rh. haemaphysaloides* showed 100% maximum identity with *E. minasensis* reported from Pakistan (OQ627010), Philippines (LC641910), Panama (OR711253), Brazil (MT212415), Australia (MH500007) and Czech Republic (JX629808), while other two dsb (336 bp) and (300 bp) sequences of *Ehrlichia* sp. from the same hosts showed 85.21% maximum identity with *Ehrlichia* sp. reported from China (CP082782), and while the other sequences showed 83.28% maximum identity with *Ehrlichia* sp. reported from China (CP082782), followed by 81–82.44% with *E. ewingii* reported from Brazil (AY428950) and USA (KM458249), and 81.72% with *Candidatus* Ehrlichia pampeana reported from Uruguay (MZ779087).

**Fig 5:**
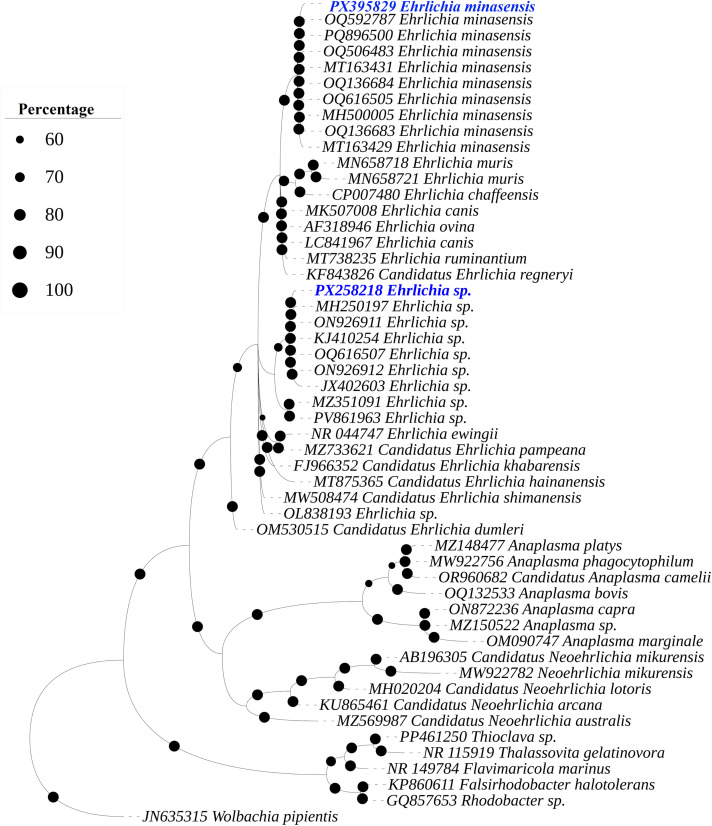
A Bayesian Inference phylogenetic tree was constructed on the basis of 16S rDNA sequences of bacterial species. The *Wolbachia pipientis* 16S rDNA sequence was used as an outgroup. The bootstrap supporting values (60-100%) are indicated at each node. The obtained sequences are represented in blue font and accession numbers: PX258218 (*Ehrlichia* sp.) and PX395829 (*Ehrlichia minasensis*).

**Fig 6:**
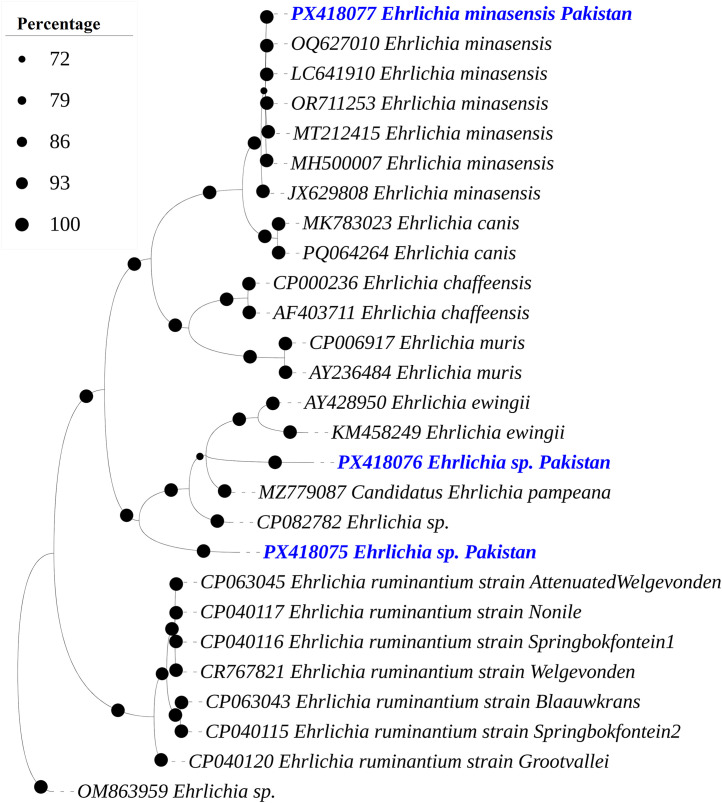
A Bayesian Inference phylogenetic tree was constructed on the basis of *dsb* sequences of *Ehrlichia* spp. The *Ehrlichia* sp. dsb sequence was used as an outgroup. The bootstrap supporting values (72-100%) are indicated at each node. The obtained sequences are represented in blue font and accession numbers: PX418075 (*Ehrlichia* sp.), PX418076 (*Ehrlichia* sp.) and PX418077 (*Ehrlichia minasensis*).

In the phylogenetic tree, the obtained 16S rDNA and *dsb* sequences of *Ehrlichia* spp. were clustered with the corresponding maximum identity sequences (Figs 5 and 6). The obtained 16S rDNA sequences for *Ehrlichia* sp. and *E. minasensis* were submitted to GenBank under the accession numbers: PX258218 and PX395829, respectively. Whereas the dsb sequences for *Ehrlichia* sp., *Ehrlichia* sp. and *E. minasensis* were submitted to GenBank under the accession numbers: PX418075, PX418076 and PX418077, respectively.

## Discussion

Ticks infesting dogs are recognized as important carriers of tick-borne microorganisms and their close association with humans and domestic animals. In Pakistan, several studies have reported the presence of ticks of different genera, including *Rhipicephalus*, infesting diverse hosts [1,3,4,26,30,31], [47–49,32,34,6,50,51]. However, data on dog-associated ticks and their role in pathogens remain limited. This study provides molecular (16S rDNA and *cox1*) evidence of *Rh. haemaphysaloides,* and *Rhipicephalus* sp., as a member of the *Rhipicephalus sanguineus* species complex, infesting stray dogs and harboring *Ehrlichia* spp. and *Ehrlichia minasensis*.

The higher infestation rates were observed in female dogs compared to males which may be due to mobility during maternal care, which increases exposure to tick infestations, which are likely consistent with previous studies [52,53]. Stray dogs with continuous outdoor exposure and a lack of veterinary care serve as ideal reservoirs for both ticks and tick-borne pathogens. These factors increase their susceptibility to tick infestations and support the maintenance of tick-borne pathogens in the environment [54,55]. However, in the present study, dogs were not examined for active infections; therefore, their role as biological reservoirs could not be directly confirmed.

The designation *Rhipicephalus* sp. reflects the ongoing taxonomic challenges associated with the *Rh. sanguineus* species complex, where closely related species exhibit similar morphological traits. Despite the description of the *Rh. sanguineus* s.s. neotype [56], misidentification within this complex remains common [57]. Molecular studies from Pakistan, have reported *Rh. sanguineus* s.l. [4,30,28,31], although uncertainties remain regarding its exact species boundaries and lineage distribution. Our study contributes further morphological and molecular evidence of this *Rh. sanguineus* species complex.

*Ehrlichia* spp. were detected in *Rhipicephalus* sp. and *Rh. haemaphysaloides* using 16S rDNA and *dsb* sequences, and phylogenetic analysis confirmed their genetic relationships with the previously reported stains. The overall prevalence observed 7.56%, which is comparable to reports from other regions where dog-associated and other ticks, such as *Rh. sanguineus, Amblyomma americanum, Dermacentor variabilis, Hyalomma dromedarii, Hyalomma marginatum, Hyalomma scupense* and *Ixodes ricinus* were screened for *Ehrlichia* spp. [16,58,59,60]. Notably, the phylogenetic analysis of the *Ehrlichia* spp. and *E. minasensis* sequences was clustered with the corresponding species sequences previously reported from Asia, Africa, Latin America, and Australia, supporting the presence of regionally circulating genetically related strains. This pattern may reflect the movement of hosts and their ticks rather than direct evidence of ehrlichiosis.

The low sequence identity (81–85%) observed in two *dsb* sequences of *Ehrlichia* sp., likely reflects the high variability of this gene among *Ehrlichia* species (76–94% interspecific) and suggesting a divergent or potentially novel lineage rather than sequence error [61,62]. This highlights the genetic diversity of *Ehrlichia* in the studied hosts and underscores the need for further multi-gene or genomic analyses to clarify their taxonomic status. The detection of *E. minasensis* previously associated with dogs and cattle, [9,63], highlights the presence of potentially pathogenic *Ehrlichia* species in ticks. Although no confirmed human cases of ehrlichiosis have been documented in Pakistan, however, the human infections caused by *E. minasensis* and *E. canis* have been reported from various countries, such as Venezuela, Panama, and northern Mexico [63,64], underscoring its zoonotic potential and possible risks to both dogs and humans. Our findings provide insights into the epidemiology of canine vector-borne diseases in Pakistan and highlight the importance of designing preventive strategies to protect both companion animals and their owners.

## Conclusion

This study reports the molecular evidence of *Ehrlichia* spp*.* and *Ehrlichia minasensis*, in *Rhipicephalus* sp. and *Rh. haemaphysaloides* ticks infesting stray dogs in northern Pakistan. The findings highlight the presence and genetic diversity of *Ehrlichia* dog associated ticks. High infestation rates and inadequate healthcare for dogs highlight the need for effective tick control, regular monitoring, and public health measures to curb tick-borne diseases. Large-scale surveillance is essential for understanding pathogen dynamics and minimizing their impact on human and animal health.
